# Sex Identification of a Multispecies Carinatae Birds by Chicken EE0.6 Gene Using Real‐Time Recombinase‐Aid Amplification Assay

**DOI:** 10.1002/ece3.70551

**Published:** 2024-11-19

**Authors:** Fanwen Zeng, Wanhuan Zhong, Tanzipeng Chen, Guoqian Wang, Jiaqi Sa, Shouquan Zhang, Hengxi Wei, Xuanjiao Chen

**Affiliations:** ^1^ Guangzhou Zoo & Guangzhou Wildlife Research Center Guangzhou China; ^2^ Kunming Institute of Zoology Chinese Academy of Sciences Kunming China; ^3^ State Key Laboratory of Swine and Poultry Breeding Industry College of Animal Science of South China Agricultural University Guangzhou China

**Keywords:** birds, EE0.6, real‐time recombinase‐aid amplification, sex identification

## Abstract

The difficulty in bird sex identification has made molecular sexing an important way to solve this problem. The conventional polymerase chain reaction (PCR) methods are time‐consuming and dependent on laboratory equipment. Recombinase‐aided amplification (RAA) is a rapid, specific, sensitive, and cost‐effective isothermal nucleic acid amplification technique. Hence, a rapid birds sexing method based on real‐time RAA targeting the unique conserved sequence 0.6‐kb EcoRI fragment (EE0.6) gene of Carinatae birds has been established and showed good specificity at 39°C for 20 min. The limit of detection for the real‐time RAA assay was determined to be 10 pg., which is 10 times more sensitive than the conventional PCR assay. For real clinical samples, the real‐time RAA assay was successfully determined sex in a subset of nine bird species and was 100% consistent with the conventional PCR assay. Consequently, the present real‐time RAA assay proves to be a powerful on‐site detection tool that can be used for an efficient and reliable birds sexing for further studies on sex ratio and captive management.

## Graphical abstract

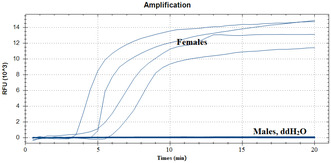

## 1 Introduction

Sex identification is valuable for estimation of the sex ratios of endangered species in wild populations and population dynamics conservation as well as evolutionary and ecological studies (Miyaki et al. 1998). However, in avian species, approximately 50% of individuals in the world are sexually monomorphic; it is often difficult to identify the sex of birds in morphology, which is especially evident in the case of nestlings (He et al. 2013; Faux, McInnes, and Jarman 2014; Ramos et al. 2009; Wang et al. 2011). Even dimorphic birds have difficulty distinguishing between the sexes by morphology when they are nestlings. At present, molecular methods have been developed and employed to sex identification in many birds, mainly through amplification and electrophoresis to analyze and determine the sequence variation between the W and Z alleles of sex‐linked chromodomain helicase DNA‐binding protein 1 (CHD1) (Ellegren 1996; Osman et al. 2020; Griffiths, Tiwari, and Becher 1992). However, the CHD1 sequences on the W and Z chromosomes are not highly conserved in many species of birds. In addition, a unique conserved sequence 0.6‐kb EcoRI fragment (EE0.6), is a non‐repetitive sequence and contains an exon‐like sequence, found on the chicken W chromosome have been utilized for Carinatae birds of sex identification (Itoh, Suzuki and Ogawa 2001). Ogawa et al. (1997) designed the USP1/USP3 primer set to successfully amplify parts of the EE0.6 sequence by polymerase chain reaction (PCR) assay in females, but not in males (Ogawa et al. 1997). Since then, many researchers have also used the EE0.6 gene to birds sexing (Kasuga et al. 2012; Kaneko et al. 2013; Insee et al. 2014; Ogawa, Murata, and Mizuno 1998). Therefore, it is a reliable method for sex determination by the difference of the EE0.6 gene on the W and Z chromosomes for many species of birds.

Among various molecular sex determination methods, PCR‐based amplification combined with gel electrophoresis is the most widely applied method (Morinha, Cabral, and Bastos 2012). Real‐time PCR combined with melt‐curve analysis (MCA) has recently been increasingly used for sex determination (Morinha et al. 2013). Although high‐resolution melting analysis is more sensitive than PCR‐based analysis, it requires expensive equipment, and is inappropriate for wide application in the field (Poojari et al. 2016; Lian et al. 2016; Teo et al. 2012). Isothermal amplification has recently been introduced for the sex identification of birds (Centeno‐Cuadros et al. 2018). Loop‐mediated isothermal amplification (LAMP) has been developed for birds sexing but requires four or more primers (Changtor, Gupta, and Yimtragool 2022). Recombinase‐based isothermal amplification assay, recombinase polymerase amplification (RPA), or recombinase‐aided amplification (RAA) is a rapid, specific, and efficient isothermal amplification technique, and can detect specific nucleic acid fragments within 10–30 min at 37°C–42°C (Tian et al. 2023; Wang, Wang and Zhang 2021; Cui et al. 2023). Compared to PCR, RPA/RAA uses recombinant enzyme, single‐stranded DNA‐binding protein (SSB), and DNA polymerase instead of the thermal cycle amplification process. This method has been widely used in the clinical detection of pathogens and sex identification (Lai et al. 2022; Cui et al. 2022; Zhang et al. 2023).

Here, we aimed to develop a novel RAA assay targeting the EE0.6 gene fragment in female birds within 20 min and then validate it using feather samples from multispecies birds. This assay provides a new, sensitive, and specific tool for the sex identification of Carinatae birds and may play an important role in on‐site detection of bird sexing.

## 2 Materials and Methods

### 2.1 Sample Collection and DNA Extraction

Feather samples from 25 bird individuals whose sexes were unknown, including domestic chicken (*Gallus gallus domesticus*), ring‐necked pheasant (*Phasianus colchicus*), golden pheasant (*Chrysolophus pictus*), Siberian grouse (*Falcipennis falcipennis*), temminck's tragopan (*Tragopan temminckii*), Chinese francolin (*Francolinus pintadeanus*), silver pheasant (*Lophura nycthemera*), wild turkey (*Meleagris gallopavo*), and Reeves's pheasant (*Syrmaticus reevesii*) were collected from Guangzhou zoo in Guangdong Province, China, in 2023 All the samples were stored at −20°C until extraction. All experimental animal protocols were reviewed and approved by the Guangzhou Zoo Animal Use and Care Committee (ID number: #YL2023002). All methods were performed in accordance with the relevant regulations and followed the recommendations outlined in the ARRIVE guidelines for conducting research.

Total genomic DNA (gDNA) was extracted following the manufacturer's protocol using HiPure Tissue & Blood DNA Kit (D3018; Megan, Guangzhou, China). After qualification and quantification using a spectrophotometer (Thermo Scientific NanoDrop, USA), the DNAs were diluted to 100 ng/μL with DNA dilution buffer (B639270‐0010; Sangon Biotech, Guangzhou, China) and stored at −20°C.

### 2.2 Design and Screening of Primers and Probes for Sex Determination

According to RAA primers and probe design guidelines and considerations, specific primer sets for the RAA assay were designed and synthesized according to the conserved EE0.6 gene sequence in *Gallus domesticus* non‐repetitive DNA sequence region from W chromosome (D85614.1). The probe was designed within the amplification region of the primer set with the highest amplification efficiency for the RAA assay The probe was labeled with FAM (6‐carboxy‐fluorescein)‐dT as a fluorophore and BHQ1 (Black Hole Quencher 1)‐dT as a quencher, while a THF site located between FAM and BHQ‐1, and had a C3 spacer at the 3′‐end block strand extension. The primers and probe specificity were assessed by the BLAST program of the National Center for Biotechnology Information (NCBI) database and then synthesized from Sangon Co. Ltd. (Shanghai, China).

### 2.3 Basic RAA Assay

Basic RAA reaction was performed using the recombinase polymerase‐based amplification kit (Hangzhou ZC, Hangzhou, China) according to the manufacturer's instructions. Briefly, the reaction mixture included 25 μL rehydration buffer, 5 μL extracted DNA template, 2 μL forward primer (10 μM), 2 μL reverse primer (10 μM), 13.1 μL ddH_2_O, and 2.5 μL magnesium acetate (280 mM). The mixture was then incubated at 39°C for 30 min. Subsequently, the products were purified using a DNA clean‐up kit (D0033, Beyotime, Shanghai, China) and subjected to electrophoresis on a 2% agarose gel and analyzed using a Bio‐Rad ChemiDoc image acquisition system.

### 2.4 Real‐Time RAA Assay

The real‐time RAA assay was performed in a 50 μL volume using the real‐time recombinase polymerase amplification kit (Hangzhou ZC) according to the manufacturer's instructions. The reaction mixture included 25 μL rehydration buffer, 5 μL extracted DNA template, 2 μL forward primer (10 μM), 2 μL reverse primer (10 μM), 0.6 μL probe (10 μM), 12.9 μL ddH_2_O, and 2.5 μL magnesium acetate (280 mM). The RAA reaction mixture was carried out at 39°C for 20 min, and was monitored using a fluorescence detector (CFX96; Bio‐Rad). A sample was considered positive when it generated an exponential amplification curve above the thereshold of the negative control.

### 2.5 Specificity of the Real‐Time RAA Assay

The specificity of the real‐time RAA assay was evaluated by testing eight *Gallus gallus domesticus* with known sex (PCR based), including four males and four females. Male DNA was used as a negative control. ddH_2_O was used as a blank control.

### 2.6 Sensitivity of the Real‐Time RAA Assay

To determine the sensitivity of the real‐time RAA assay for the EE0.6 gene sequence in *Gallus gallus domesticus* non‐repetitive DNA sequence region from W chromosome, the genomic DNA of female *Gallus gallus domesticus* was 10‐fold serially diluted using DNA dilution buffer from 100 ng to 10 fg and conducted for eight independent repeats, with ddH_2_O as blank control.

PCR reactions were carried out in a 20 μL volume comprising 2 μL genomic DNA, 1 μL each primer (10 μM), 10 μL 2× Dream*Taq* PCR Master Mix polymerase (K1081, Thermo Fisher Scientific, Massachusetts, USA), and 4 μL ddH_2_O. Primers used were USP1/USP3 (USP1: 5‐CTATGCCTACCACMTTCCTATTTGC‐3; USP3: 5‐AGCTGGAYTTCAGWSCATCTTCT‐3) and SINT‐F/SINT‐R (SINT‐F: 5‐TAGGCTGCAGAATACAGCAT‐3; SINT‐R: 5‐TTGTGCAGTTCTAGTCCATA‐3) (Ogawa et al. 1997). The amplification protocol consisted of the following steps: initial denaturation at 94°C for 5 min, followed by 35 cycles of denaturation at 94°C for 50 s, annealing at 51.5°C for 50 s, extension at 72°C for 50 s, and then a final extension at 72°C for 5 min. The PCR products were separated by electrophoresis for 30 min on a 2% agarose gel and visualized using an ultraviolet transilluminator (Bio‐Rad, California, USA).

### 2.7 Validation Test of the Real‐Time RAA Assay

The diagnostic applicability of the real‐time RAA assay for sex identification of birds was evaluated by testing 25 individuals of 9 species with unknown sex and comparing it with a conventional PCR assay. Samples were obtained from the Guangzhou Zoo in Guangdong province, China. All procedures of the real‐time RAA assay were carried out in accordance with the above‐described conditions.

### 2.8 Statistical Analysis

For the limit of detection data of the real‐time RAA assay, a probit regression analysis and a semi‐log regression analysis were conducted using GraphPad Prism 8.0 software (GraphPad Software, California, USA). The data are represented as the mean.

## 3 Results

### 3.1 Screening of the Primer Sets

**FIGURE 1:**
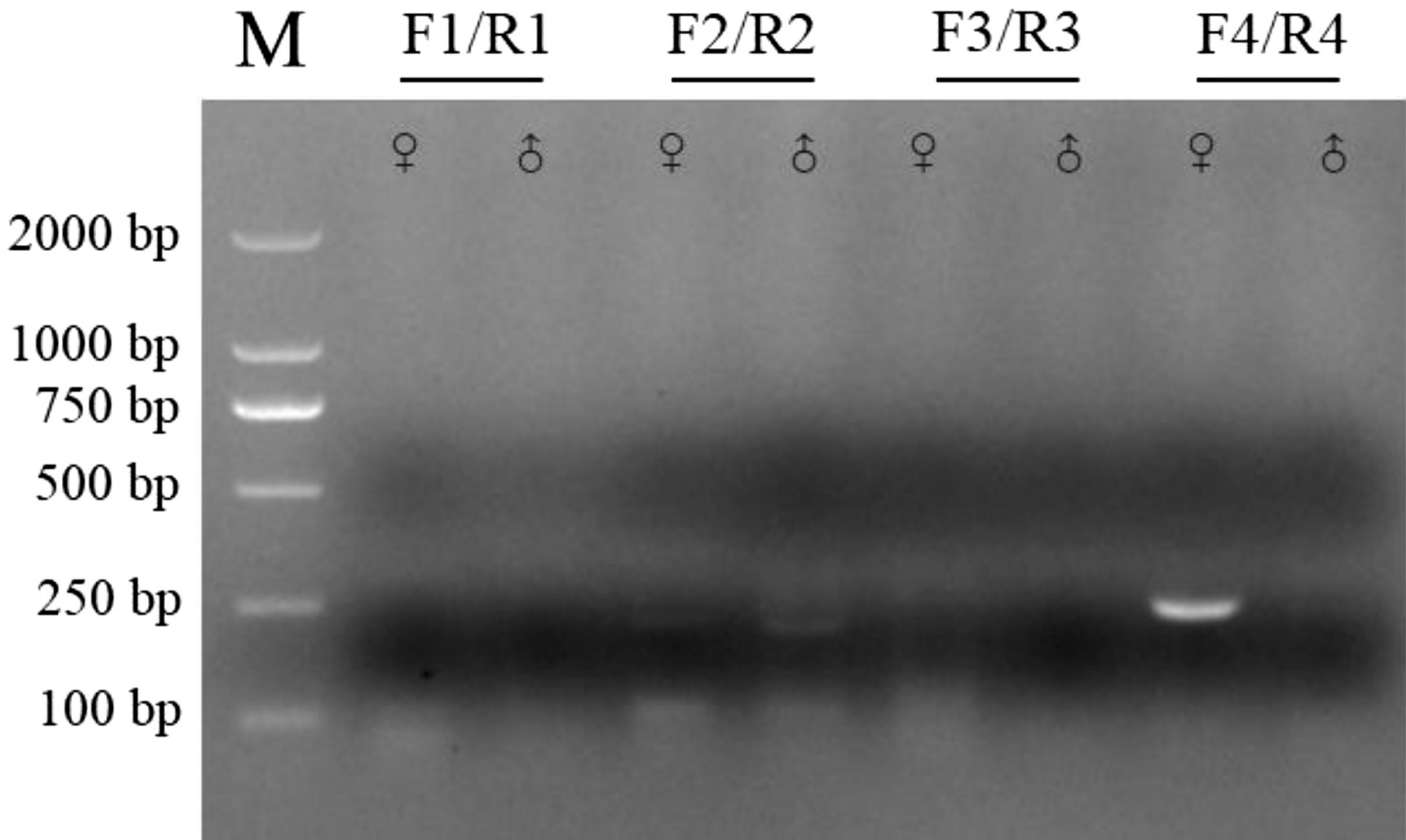
Primer sets screening for the basic recombinase‐aided amplification (RAA) assay. The RAA amplifies products using four primer sets were subjected to electrophoresis on a 2% agarose gel, respectively. M: DL 2000 DNA Marker.

Four primer sets were designed for the development of the EE0.6‐specific real‐time RAA assay (Table 1). To screen the optimal primer set, we used the basic RAA assay combined with electrophoresis on agarose gel. As shown in Figure 1, the primer set F4/R4 successfully amplified the target region of female, but not of male. Subsequently, we designed the probe based on the amplification region of the F4/R4 primer set. The details of primers and probe used in this study are shown in Table 1.

**TABLE 1:** Sequences of the primers and internal probe used for the RAA assay in this study.

Primers/Probes	Sequence (5′–3′)	Genomic position
EE0.6‐raa‐F1	ACTTGAGATAGTGACTTCCTTCTGGCAAAG	34–63
EE0.6‐raa‐R1	CCATCTTCTAAGTAATGGGTTCAGGTTGAG	430–459
EE0.6‐raa‐F2	TAGCAGTTATGGTCCTATGCCTACCACATT	86–115
EE0.6‐raa‐R2	TTGTTGGCACTGATTTAGTTCCTTGCTTGT	317–346
EE0.6‐raa‐F3	AGGGAGTATCTAGCAGTTATGGTCCTATGC	76–105
EE0.6‐raa‐R3	CCATCTTCTAAGTAATGGGTTCAGGTTGAG	430–459
EE0.6‐raa‐F4	TTATGGTCCTATGCCTACCACATTCCTATT	92–121
EE0.6‐raa‐R4	TGTTGTTGGCACTGATTTAGTTCCTTGCTT	319–348
EE0.6‐raa‐probe	ACATTAGGGTCACTGAATTTTACTTAAAAG (FAM‐dT) (THF) (BHQ1‐dT) CAGTGCATTTATTTT‐C3 spacer	187–234

### 3.2 Specificity of the Real‐Time RAA Assay

**FIGURE 2:**
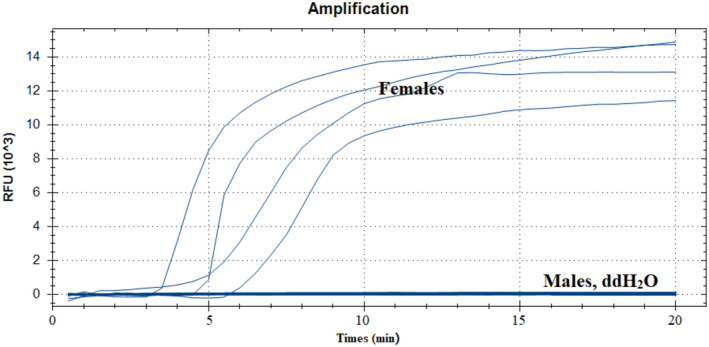
Specificity test results of the real‐time recombinase‐aided amplification (RAA) assay. The RAA reactions were incubated at 39°C for 20 min. Males: Negative control.

To evaluate the specificity of the real‐time RAA assay, female and male samples were evaluated. The results showed (Figure 2) that all females gDNA could generate remarkable fluorescence signals, but none of the males gDNA, indicating that the primers and probe had no cross‐reactivity with males and had good specificity.

### 3.3 Limit of Detection of the Real‐Time RAA Assay

**FIGURE 3:**
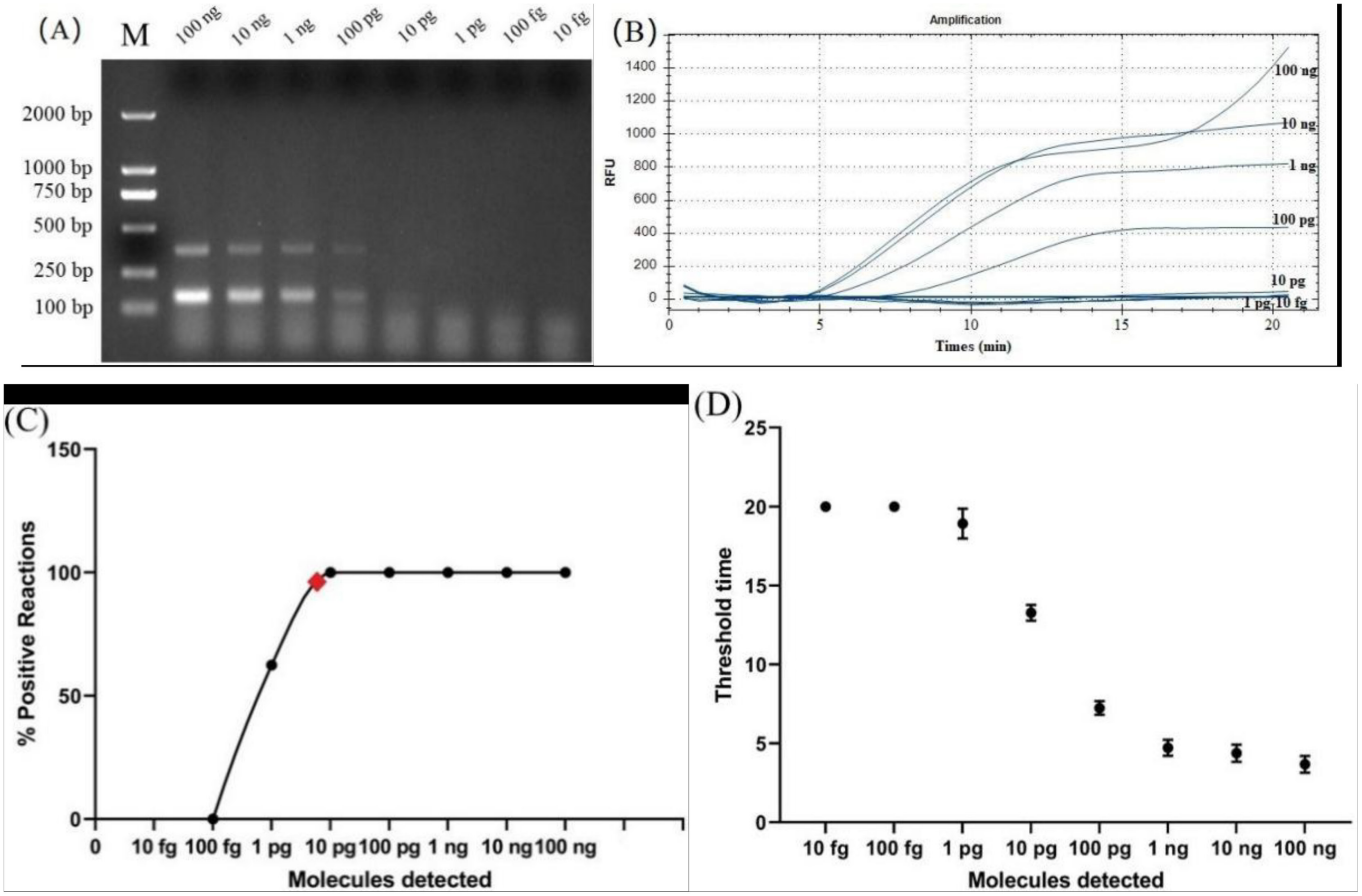
Sensitivity evaluation of recombinase‐aided amplification (RAA) and PCR assays. (A) Limit of detection of the conventional PCR assay run with USP1/SUP3 and SINT‐F/SINT‐R primer sets. (B) The results of real‐time RAA with different concentrations (100 ng^−1^pg) of female *Gallus gallus domesticus* DNA. The RAA reactions were incubated at 39°C for 20 min. (C) Probit regression analysis of the data collected from the eight real‐time RAA repeats using GraphPad Prism 8.0 software. The limit of detection at 95% probability (10 pg/reaction) is depicted by a red rhomboid. (D) Semi‐logarithmic regression of the data collected from the eight real‐time RAA repeats using GraphPad Prism 8.0 software. The data were represented as the mean.

The sensitivity of the real‐time RAA assay and PCR assay was assessed by conducting tests on 10‐fold serial dilutions ranging from 100 ng to 0.1 fg of genomic DNAs from female pheasant. The results showed that the analytical sensitivity of real‐time RAA was found to be 10 pg and PCR demonstrated a detection limit of 100 pg (Figure 3A,B). A probit regression analysis was performed for eight replicates of data and a detection limit of 10 ng/reaction in 95% of cases (Figure 3C). Semi‐log regression analysis indicated that the reaction time lengths of the real‐time RAA assay were 2.6–14 min for 100 ng to 10 pg of gDNA (Figure 3D).

### 3.4 Reliability of the Real‐Time RAA Assay

The accuracy and application of the real‐time RAA assay in sex determination was assessed for 25 individuals. The detection results of real‐time RAA and the PCR results are shown in Table 2. These results indicated that the real‐time RAA assay fully matched PCR‐based sex determination and was effective for real clinical samples.

**TABLE 2:** Comparative reliability of real‐time RAA and conventional PCR assays for birds sexing.

No.	Sample species	Real‐time RAA	Conventional PCR		
		Female	Male	Female	Male
1	*Gallus gallus domesticus*	+	−	+	−
2	*Gallus gallus domesticus*	+	−	+	−
3	Gallus gallus *domesticus*	−	+	−	+
4	*Phasianus colchicus*	+	−	+	−
5	*Phasianus colchicus*	−	+	−	+
6	*Chrysolophus pictus*	−	+	−	+
7	*Chrysolophus pictus*	−	+	−	+
8	*Chrysolophus pictus*	−	+	−	+
9	*Chrysolophus pictus*	+	−	+	−
10	*Falcipennis falcipennis*	+	−	+	−
11	*Falcipennis falcipennis*	−	+	−	+
12	*Tragopan temminckii*	+	−	+	−
13	*Tragopan temminckii*	−	+	−	+
14	*Francolinus pintadeanus*	+	−	+	−
15	*Francolinus pintadeanus*	+	−	+	−
16	*Francolinus pintadeanus*	−	+	−	+
17	*Francolinus pintadeanus*	−	+	−	+
18	*Lophura nycthemera*	+	−	+	−
19	*Lophura nycthemera*	−	+	−	+
20	*Meleagris gallopavo*	+	−	+	−
21	*Meleagris gallopavo*	−	+	−	+
22	*Syrmaticus reevesii*	+	−	+	−
23	*Syrmaticus reevesii*	+	−	+	−
24	*Syrmaticus reevesii*	+	−	+	−
25	*Syrmaticus reevesii*	−	+	−	+

## 4 Discussion

Accurate sexing is a critical information for captive management and selection of the effective population for reintroduction programs in any effective conservation program (Griffiths and Tiwari 1993; Turcu et al. 2023). Molecular sexing in birds is considered one of the most reliable methods (England et al. 2021). The RAA assay is much more rapid than any other existing technique, completing the amplification of the target sequence in 10–20 min with a high specificity and sensitivity. Additionally, RAA is suitable for rapid application and efficient field research (Fan et al. 2020; Chen et al. 2022; Zhao et al. 2021). This study describes the development and evaluation of a real‐time RAA assay for sex identification of birds. The real‐time RAA method finishes in 20 min, was developed by identifying the EE0.6 gene of sex chromosomes W, which can successfully differentiate males from females and is suitable for a wide variety of birds.

The EE0.6 sequence is widely conserved in several species of birds and is present only on the female‐specific W chromosome (Ogawa et al. 1997). The rest of the genes that have been found on the chicken W chromosome have their counterpart genes on the Z chromosome (Tagliavia et al. 2023; Bantock, Prys‐Jones, and Lee 2008; O'Neill et al. 2000) Itoh, Hori and Saitoh 2001. Therefore, the EE0.6 sequence seems to have certain advantages as a target for sex determination. At present, many birds, including *Grus antigone sharpii*, *Ciconia boyciana*, and *Nipponia nippon*, have been sex determined by the PCR method based on the EE0.6 gene sequence (Kasuga et al. 2012; Insee et al. 2014; Itoh et al. 1997). In this study, we used the EE0.6‐RAA‐F4/R4 and probe combination designed based on the amplification of the female‐specific gene sequence EE0.6 on the W chromosome to achieve 100% success in sex identification of a variety of birds. However, we found that there are still some birds that cannot be sexed using this method because the EE0.6 gene sequence is not consistent in all birds. Therefore, we still need to further improve the method.

The conventional sex identification methods for birds are predominantly invasive and cause great harm to individuals. To address this problem, PCR assays have been widely used for sex determination of birds. Most of the time, we can only collect feathers for identification, which requires high sensitivity of primers. It is known that the sensitivity of the RAA method is higher than that of the PCR method (Lv et al. 2022; Wu et al. 2021; Wang, Li and Lin 2021). The present finding that the real‐time RAA method based on the EE0.6 gene showed good detection sensitivity, is 10 times more sensitive than the PCR method by USP1/USP3 and SINT‐F/SINT‐R primer sets. Only a feather sample is needed to complete the sex determination, without causing harm to the individual. The PCR method has the possibility that the analysis of the amplification products was performed by gel electrophoresis, which not only affected the sensitivity but also limited the detection assay to the laboratory (Ma et al. 2021).

In essence, isothermal amplification methods, either LAMP or RPA/RAA, were developed for field research. As reported, (Lai et al. 2022) designed a pair of primers and probes based on the CHD gene, and used RPA to quickly identify the sex of pigeons in the field. (Zhao et al. 2021) established a novel RAA visual system to identify the genetic sex of *Cynoglossus semilaevis* in the field. This is also true for the real‐time RAA method of this study, and this study can be used for bird sex identification in field studies. However, in this study, simplified DNA extraction for the field test has yet to be established, and this will be a focal point of the focus and direction of our future research.

In conclusion, we successfully developed a simple, specific, and sensitive real‐time RAA method based on the EE0.6 sequence for sex identification of multispecies Carinatae birds. It is an efficient and reliable on‐site detection tool for a feasible alternative to laboratory methods.

## Author Contributions

**Fanwen Zeng:** writing – original draft (lead). **Wanhuan Zhong:** data curation (equal). **Tanzipeng Chen:** investigation (equal), validation (equal). **Guoqian Wang:** software (equal), validation (equal). **Jiaqi Sa:** formal analysis (equal), visualization (equal). **Shouquan Zhang:** methodology (equal). **Hengxi Wei:** conceptualization (equal). **Xuanjiao Chen:** project administration (equal), supervision (equal).

## Conflicts of Interest

The authors declare no conflicts of interest.

## Supporting information


Appendix S1

Appendix S1

## Data Availability

All data are included in this manuscript.
