# Supplementary material for: Sex Identification of a Multispecies Carinatae Birds by Chicken EE0.6 Gene Using Real‐Time Recombinase‐Aid Amplification Assay
Source: Ecol Evol. 2024 Nov 19;14(11):e70551. doi: 10.1002/ece3.70551 (PMC11575936; doi:10.1002/ece3.70551)
Supplement: Supplementary file 1 — Appendix S1 [file ECE3-14-e70551-s002.docx]

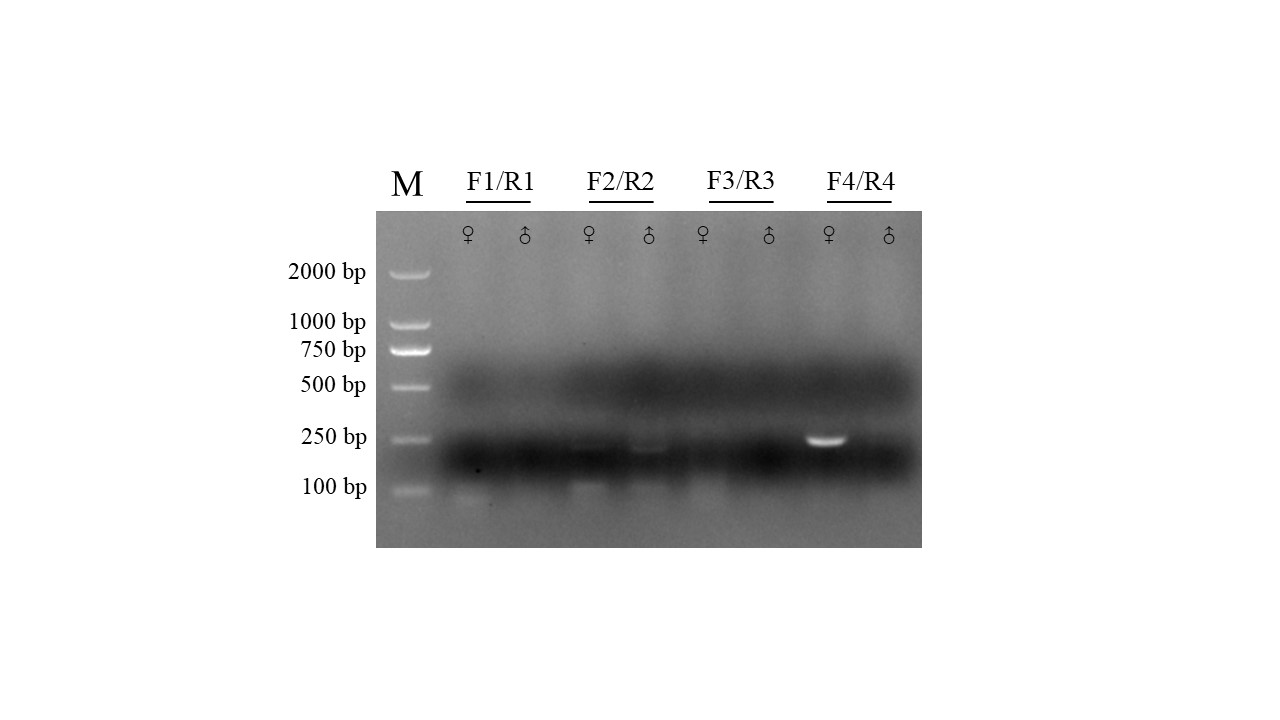


**Figure.1 Primer sets screening for basic recombinase-aided amplification (RAA) assay.** The RAA amplify products using four primer sets were subjected to electrophoresis on a 2% agarose gel respectively. M: DL 2000 DNA Marker.


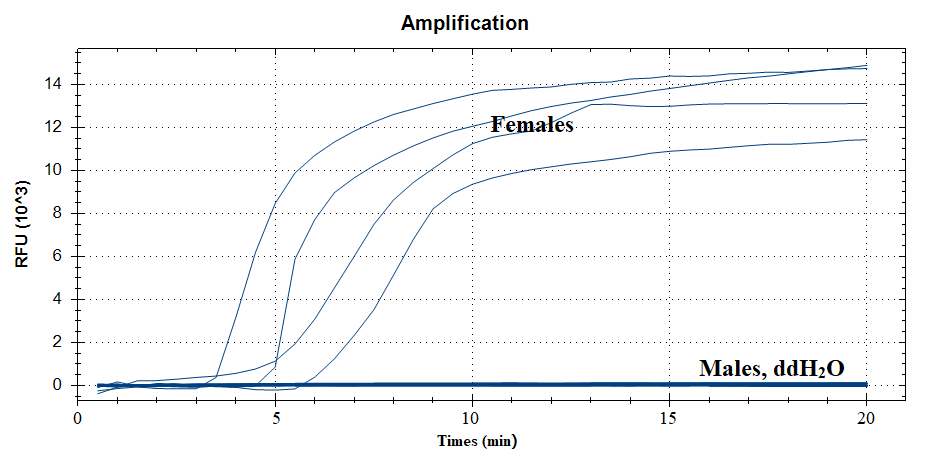


**Figure.2 Specificity test results of real-time recombinase-aided amplification (RAA) assay.** The RAA reactions incubated at 39℃ for 20 min. Males: Negative control.


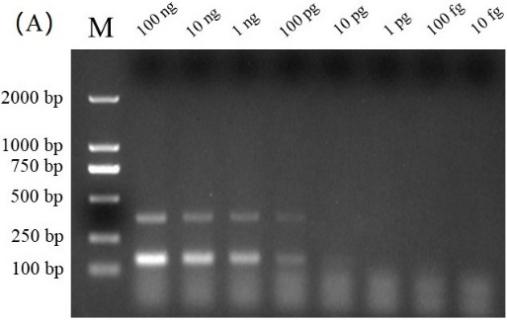

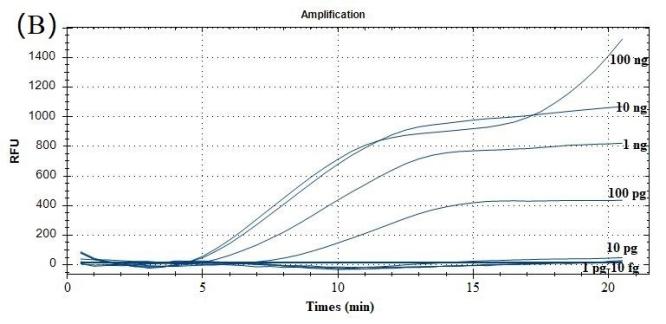


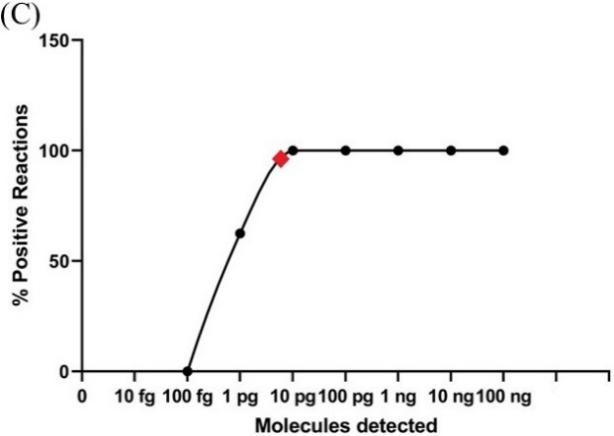

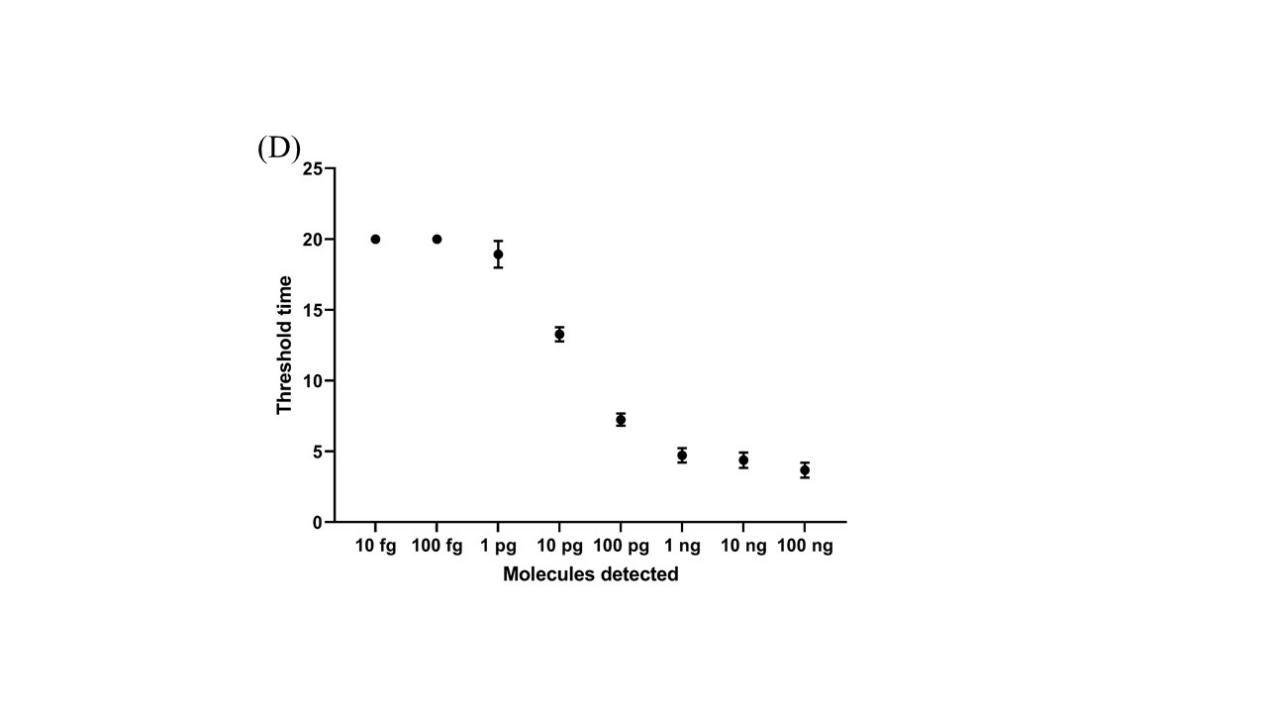


**Figure.3 Sensitivity evaluation of recombinase-aided amplification (RAA) assay and PCR assay**. **(A)** Limit of detection of conventional PCR assay run with USP1/SUP3 and SINT-F/SINT-R primer sets. **(B)** The results of real-time RAA with different concentrations (100 ng-1 pg) of female gallus domesticus DNA. The RAA reactions incubated at 39℃ for 20 min. **(C)** Probit regression analysis of the data collected from the eight real-time RAA repeats using GraphPad Prism 8.0 software. The limit of detection at 95% probability (10 pg/reaction) is depicted by a red rhomboid. **(D)** Semi-logarithmic regression of the data collected from the eight real-time RAA repeats using GraphPad Prism 8.0 software. The data was represented as the mean.
