# Supplementary material for: Sex Identification of a Multispecies Carinatae Birds by Chicken EE0.6 Gene Using Real‐Time Recombinase‐Aid Amplification Assay
Source: Ecol Evol. 2024 Nov 19;14(11):e70551. doi: 10.1002/ece3.70551 (PMC11575936; doi:10.1002/ece3.70551)
Supplement: Supplementary file 2 — Appendix S1 [file ECE3-14-e70551-s001.docx]

**Table 1. Sequences of the primers and internal probe used for the RAA assay in this study.**

| Primers/Probe | Sequence (5'–3') | Genomic position |
| --- | --- | --- |
| EE0.6-raa-F1 | ACTTGAGATAGTGACTTCCTTCTGGCAAAG | 34-63 |
| EE0.6-raa-R1 | CCATCTTCTAAGTAATGGGTTCAGGTTGAG | 430-459 |
| EE0.6-raa-F2 | TAGCAGTTATGGTCCTATGCCTACCACATT | 86-115 |
| EE0.6-raa-R2 | TTGTTGGCACTGATTTAGTTCCTTGCTTGT | 317-346 |
| EE0.6-raa-F3 | AGGGAGTATCTAGCAGTTATGGTCCTATGC | 76-105 |
| EE0.6-raa-R3 | CCATCTTCTAAGTAATGGGTTCAGGTTGAG | 430-459 |
| EE0.6-raa-F4 | TTATGGTCCTATGCCTACCACATTCCTATT | 92-121 |
| EE0.6-raa-R4 | TGTTGTTGGCACTGATTTAGTTCCTTGCTT | 319-348 |
| EE0.6-raa-probe | ACATTAGGGTCACTGAATTTTACTTAAAAG (FAM-dT)(THF)(BHQ1-dT) CAGTGCATTTATTTT-C3 spacer | 187-234 |

**Table 2. Comparative reliability of real-time RAA and conventional PCR assays for birds sexing.**

| **No.** | **Sample species** | **Real-time RAA** | | | **Conventional PCR** | | | |
| --- | --- | --- | --- | --- | --- | --- | --- | --- |
|  |  | female | male | | | female | male | |
| 1 | *Gallus gallus domesticus* | + | | - | | + | | - |
| 2 | *Gallus gallus domesticus* | + | | - | | + | | - |
| 3 | *Gallus gallus domesticus* | - | | + | | - | | + |
| 4 | *Phasianus colchicus* | + | | - | | + | | - |
| 5 | *Phasianus colchicus* | - | | + | | - | | + |
| 6 | *Chrysolophus pictus* | - | | + | | - | | + |
| 7 | *Chrysolophus pictus* | - | | + | | - | | + |
| 8 | *Chrysolophus pictus* | - | | + | | - | | + |
| 9 | *Chrysolophus pictus* | + | | - | | + | | - |
| 10 | *Falcipennis falcipennis* | + | | - | | + | | - |
| 11 | *Falcipennis falcipennis* | - | | + | | - | | + |
| 12 | *Tragopan temminckii* | + | | - | | + | | - |
| 13 | *Tragopan temminckii* | - | | + | | - | | + |
| 14 | *Francolinus pintadeanus* | + | | - | | + | | - |
| 15 | *Francolinus pintadeanus* | + | | - | | + | | - |
| 16 | *Francolinus pintadeanus* | - | | + | | - | | + |
| 17 | *Francolinus pintadeanus* | - | | + | | - | | + |
| 18 | *Lophura nycthemera* | + | | - | | + | | - |
| 19 | *Lophura nycthemera* | - | | + | | - | | + |
| 20 | *Meleagris gallopavo* | + | | - | | + | | - |
| 21 | *Meleagris gallopavo* | - | | + | | - | | + |
| 22 | *Syrmaticus reevesii* | + | | - | | + | | - |
| 23 | *Syrmaticus reevesii* | + | | - | | + | | - |
| 24 | *Syrmaticus reevesii* | + | | - | | + | | - |
| 25 | *Syrmaticus reevesii* | - | | + | | - | | + |
